# Recovery Strategies for Heavy Metal-Inhibited Biological Nitrogen Removal from Wastewater Treatment Plants: A Review

**DOI:** 10.3390/microorganisms10091834

**Published:** 2022-09-14

**Authors:** Ncumisa Mpongwana, Sudesh Rathilal, Emmanuel K. Tetteh

**Affiliations:** Green Engineering Research Group, Department of Chemical Engineering, Faculty of Engineering and the Built Environment, Durban University of Technology, Durban 4000, South Africa

**Keywords:** annamox, bio-accelerator, inhibition, heavy metals, wastewater

## Abstract

Biological nutrient removal is an integral part of a wastewater treatment plant. However, the microorganism responsible for nutrient removal is susceptible to inhibition by external toxicants such as heavy metals which have the potential to completely inhibit biological nutrient removal. The inhibition is a result of the interaction between heavy metals with the cell membrane and the deoxyribonucleic acid (DNA) of the cell. Several attempts, such as the addition of pretreatment steps, have been made to prevent heavy metals from entering the biological wastewater systems. However, the unexpected introduction of heavy metals into wastewater treatment plants result in the inhibition of the biological wastewater treatment systems. This necessitates the recovery of the biological process. The biological processes may be recovered naturally. However, the natural recovery takes time; additionally, the biological process may not be fully recovered under natural conditions. Several methods have been explored to catalyze the recovery process of the biological wastewater treatment process. Four methods have been discussed in this paper. These include the application of physical methods, chelating agents, external field energy, and biological accelerants. These methods are compared for their ability to catalase the process, as well as their environmental friendliness. The application of bio-accelerant was shown to be superior to other recovery strategies that were also reviewed in this paper. Furthermore, the application of external field energy has also been shown to accelerate the recovery process. Although EDTA has been gaining popularity as an alternative recovery strategy, chelating agents have been shown to harm the metal acquisition of bacteria, thereby affecting other metabolic processes that require heavy metals in small amounts. It was then concluded that understanding the mechanism of inhibition by specific heavy metals, and understanding the key microorganism in the inhibited process, is key to developing an effective recovery strategy.

## 1. Introduction

A wide range of toxic contaminants are being increasingly introduced into the environment due to the increase in industrialization, which has raised many concerns. Heavy metals are one of the contaminants of concern since they result in serious damage to the environment [1]. These concerns have increased, thereby increasing interest in studies that seek to develop effective technologies for the elimination of these contaminants from the environment. If these contaminants are left untreated, they end up in water streams through various pathways such as run-offs and industrial waste [2,3]. The most toxic heavy metals have been reported to be the metals arsenic, cadmium, and lead [4]. These heavy metals have been said to be carcinogenic and can result in serious health hazards if left untreated. These metals affect many biological processes in the environment and can inhibit biological processes when they enter the wastewater treatment plant (WWTP) [4,5].

Anammox, nitrification, and anaerobic denitrification have been reported to be the processes that can be completely inhibited by heavy metal loadings in WWTPs. Nitrification involves the use of ammonia oxidizing bacteria (AOB) that convert nitrogen into nitrite, while denitrification involves the use of nitrite-oxidizing bacteria (NOB) to convert nitrite to nitrate. AOB are known to be sensitive and easily inhibited by a wide range of toxic contaminant loadings [6]. The inhibition of these processes may result in the disposal of effluent with total nitrogen exceeding the acceptable discharge limits. Moreover, discharging of effluent with high nitrogen content may prohibit the re-utilization of treated water for recreational activities. Research investigating strategies to recover nitrification and anaerobic denitrification after inhibition by heavy metals has gained attention over the years [7], with many scholars investigating techniques that have a shorter recovery time. Traditional methods for heavy metal removal such as chemical oxidation or reduction, ion exchange, electrochemical treatment, reverse osmosis, and evaporation have been used as a strategy to recover the inhibited nitrification and anaerobic denitrification [8].

However, these methods are not always effective, particularly for unexpected inhibition of activated sludge. Additionally, these methods may result in the production of toxic byproducts that may cause further damage to bacterial cells, thus making it even more difficult to recover nitrification and anaerobic denitrification. Furthermore, these processes are not always applicable owing to their expensiveness [8]. The role of the chelating agent has also been investigated for the recovery of inhibited nitrification and anaerobic denitrification. EDTA is one of the popular chelating agents which have been investigated thus far. It was shown in some studies that EDTA decreases the inhibition exacted by toxic contaminants on biological nutrient removal bacteria. However, the drawback of applying EDTA for the recovery of nitrification and anaerobic denitrification is that EDTA impairs the nitrification process [8]. This has resulted in rising interest in the application of bio-accelerators to recover nitrification and anaerobic denitrification. Bio-accelerators have been noted to be an environmentally friendly and cost-effective way of enhancing the recovery of nitrification and anaerobic denitrification after heavy metal inhibition [7,9].

Several bio-accelerators have been shown to promote recovery of nitrification and anaerobic denitrification. Among many, Biotin (Vitamin H) has been found to enhance biochemical oxygen demand (BOD) and chemical oxygen demand (COD) removal, thereby recovering nitrification and anaerobic denitrification. Flavin adenine dinucleotide (FAD) is another bio-accelerator that has been reported to effectively enhance microbial growth, thus promoting the recovery of biological wastewater treatment [7]. Wang et al. [9] investigated the recovery of denitrifying bacteria using biotin, L-aspartic acid, and cytokinin accelerators. The authors found that nitrification was recovered within 8 days when combining these bio-accelerators. Although there has been an increase in studies seeking to improve the recovery process of biological wastewater treatment, with many studies reporting potential alternatives, to our knowledge there is no literature comparing these methods. Hence, this paper aims to review different methods for recovery of nitrification and anaerobic denitrification. Moreover, an in-depth analysis of each method, drawbacks, benefits, and gaps in the literature will be highlighted.

## 2. Methods for Biological Nitrogen Removal

### 2.1. Nitrification and Denitrification

In WWTPs the traditional method of treating total nitrogen is through nitrification and denitrification, for which the first step is ammonium oxidation which depends on autotrophic nitrifying bacteria (ANB) [10]. The second step, which is denitrification, occurs under anoxic conditions [11]. Due to contrasting operation requirements for this process, they occur in separate reactors. However, the use of multiple reactors is expressive. Hence, there has been a rising interest in simultaneous nitrification and denitrification (SND); in SND, both nitrifications occur in a single reactor [12]. Recently, several studies have shown the possibility of simultaneous nitrification and denitrification (SND) for total nitrogen removal by denitrifying glycogen-accumulating organisms (DGAOs). The use of DGAO is advantageous due to its low requirement for external organic matters. Phosphorus removal is carried out by polyphosphate accumulating organisms (PAOs) or denitrifying PAOs (DPAOs). Separate N and P removal processes may require high energy for aeration, and 60% of the total energy consumption in WWTPs is used for aeration of these processes. Interestingly both SND and biological phosphorus removal (EBPR) occur under anoxic conditions, thus SND and EBPR can be performed in the same reactor. Combining these processes in one reactor can potentially reduce aeration requirements and reduce up to 50% of carbon requirements because both P-removal and denitrification use the same electron donor when performed simultaneously. This in turn can reduce the cost of N and P removal [13].

### 2.2. Anammox

Due to the high energy required for aeration of traditional aerobic and anaerobic denitrification, and the requirement of organics addition for the treatment of high-strength ammonium in WWTPs, an alternative method for nitrogen removal was designed. This method is known as anaerobic ammonium oxidation (anammox). Anammox addresses the drawbacks of traditional aerobic nitrification and anaerobic denitrification [14]. Anammox transforms NH_4_^+^ into dinitrogen (N_2_) gas, using NO_2_^−^ as an electron acceptor. This process occurs under anaerobic conditions. This process decreases oxygen demand by 60% from oxygen demand (aeration) required in the traditional aerobic nitrification and anaerobic denitrification and reduces the sludge generation by 90%. Nitrogen removal through anammox occurs in two steps, with partial nitrification being the first step. In this step only half of NH_4_^+^ gets oxidized to NO_2_^−^. The second step is an anammox process where NO_2_^−^ is used as an electron acceptor for NH_4_^+^ oxidation to N_2_ under anoxic conditions. This process is known as partial nitrification and anammox (PN&A). Traditionally, PN&A is carried out in two separate stages. However, these processes can occur in a single reactor [15,16]. Anammox reduces the cost of N removal as compared to traditional aerobic nitrification and anaerobic denitrification. A survey conducted in Germany showed that anammox saves up to 83.5% of costs compared to traditional aerobic nitrification and anaerobic denitrification [14].

### 2.3. Nutrient Removal by Algae/Microalgae-Based Processes

High concentrations of heavy metals are not only detrimental to conventional biological processes, but they are also toxic to Phycoremediation which is the process that uses algae for the treatment of water. Microalgae uses the nutrient in wastewater for proliferation, which in turn provides photo-oxygenation of the waters [17]. High-rate algal ponds (HRAP) have gained popularity in recent years and may be potential alternatives to conventional biological wastewater treatment technology due to their low aeration equipment. Moreover, the main reason microalgae is preferred for the treatment of wastewater is that they can be reused and recycled since, after use, they become nutrient-rich and suitable to be used as feedstock for polymers, biogas or biodiesel, and the production of fertilizer [18]. Furthermore, microalgal applications in wastewater have attracted attention since they can accumulate toxicants that would otherwise be toxic to conventional biological wastewater treatment plants such as heavy metals, pesticides, and organic and inorganic toxic compounds. Other benefits of microalgae include low energy requirements, cost-effectiveness, and low sludge and biomass production [19,20]. Although several heavy metals such as Mn^2+^, Ni^2+^, Cu^2+^, Mo^2+^, Fe^2+^, and Zn^2+^ are vital for the growth of algae, certain heavy metals are toxic to algal growth such as Sn^2+^, Au^3+^, Cd^2+^, Pb^2+^, Sr^2+^, Ti^3+^, and Hg^2+^. These metals have no vital biological function to algae. Although algae have been shown to have a tolerance to heavy metals, these heavy metals may negatively impact the growth of algae. Thus, they affect the removal of toxins since the removal efficiency of dead algal is lower than that of a living cell. This is attributed to the fact that living cells use both biosorption and bioaccumulation, unlike dead cells, which only use biosorption to remove heavy metals [21].

## 3. Inhibition of Biological Nitrogen Removal Process by Heavy Metals

**Figure 1:**
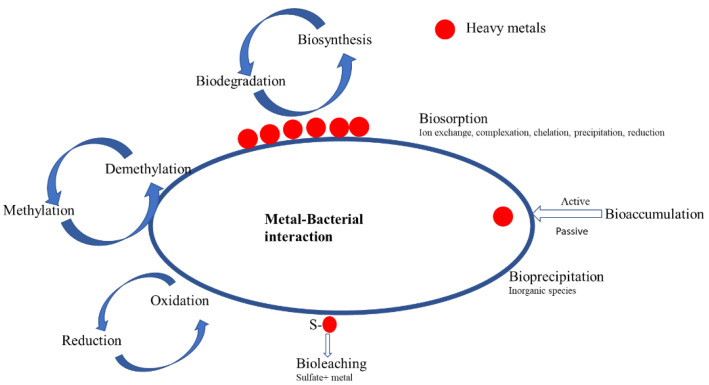
Diagram depicting interaction between bacteria and metal species edited from [26].

Aerobic nitrification and anaerobic denitrification play a crucial role in WWTPs, although these processes are effective in biological nitrogen removal (BNR). They are susceptible to inhibition by toxic contaminants that enter WWTPs, and heavy metals are among contaminants known to inhibit aerobic nitrification and anaerobic denitrification. The heavy metals which have been reported to possess a high inhibition effect on biological processes include Ni (II), Zn (II), Cd (II), and Pb (II); these heavy metals enter the municipality WWTPs through discharge from industries. AOB is the most sensitive bacteria to heavy metal loadings, and the presence of high heavy metal loading in WWTPs affects the microbial metabolism by altering the regulation of enzymes responsible for the nitrification process. For example, Cd (II) reduces the expression of ammonia monooxygenase (amoA) and therefore decreases the ammonia oxidation activity of *Nitrosomonas europaea*, while Zn (II) may result in up-regulation of amoA and does not significantly affect the expression of Hydroxylamine oxidoreductase (hao). Additionally, the changes in microbial metabolism are also linked with changes in the expression of nitrite reductase (NirK) and nitric oxide reductase (NorB), which are the gene coding for autotrophic nitrite and nitric oxide reduction, respectively [22]. Heavy metals have also been found to inhibit anammox [8]. Kimura and Isaka [23] investigated the impact of Ni, Cu, Co, Zn, and Mo on anammox, and the result obtained from this study indicated that 5, 5, 5, 10, and 0.2 mg/L, respectively of these metals resulted in a 10% decrease in anammox. Moreover, the authors further noted that inhibition caused by Ni, Cu, Co, and Zn was reversible, while inhibition caused by Mo on anammox was not reversible. Additionally, Gutwiński et al. [24] and Zhang et al. [25] also reported inhibition of anammox by various heavy metals. Figure 1 shows different types of microbial interactions between heavy metals and microorganisms. The inhibition of biological processes by heavy metals occurs because of metal uptake, extracellular sorption, transmembrane transport, and intracellular accumulation by microorganisms. Sorption occurs because of the binding sites such as amino, carboxylic, hydroxyl, and phosphate functional groups that are available on the surface of the microorganism. The mechanism of cell inhibition begins with the diffusion of metal across the outer walls via porins and moves into the cytoplasmic membrane where it then interacts with nucleic acid and active sites of enzymes. This results in a decrease in membrane integrity, thereby leading to the leakage of cellular solutes such as K^+^ after cell death (Figure 1).

## 4. Mechanism of Heavy Metal Inhibition of Biological Methods

The mechanism by which each heavy metal inhibits the biological process is important, especially for designing the recovery strategy. Hence, it is crucial to discuss these mechanisms. Therefore, this section will discuss the mechanism of biological inhibition by heavy metals.

### 4.1. Copper

High concentrations of soluble Cu (II) are toxic to microorganisms. It affects many biological processes in wastewater treatment, and BNR is no exception. As low as 1.9 mg/L of Cu (II) can result in up to a 50% decrease of Anammox bacteria in a batch system [27]. The inhibition of Cu (II) in the biological system is attributed to its antimicrobial action and the impact it has on cell metabolism (refer to Table 1). Generally, copper is commonly used as the main ingredient in bactericides and fungicides. The antimicrobial activity of Cu (II) is due to its ability to chelate sulfhydryl groups, which results in the destruction of enzymes and cell proteins. The inhibition potential of Cu (II) in wastewater treatment has been reported to result from sulfate reduction in aerobic heterotrophic bacteria [28]. The metabolic destruction of Cu (II) towards anammox activity is due to its impact on autotrophic anaerobic ammonium oxidizing bacteria (AnAOB), which are highly sensitive to Cu (II). The interaction between Cu (II) and AnAOB has been studied and the mechanism in which Cu (II) and AnAOB interact is said to be important and can potentially provide direction for remedial strategies to prevent inhibition of biological systems by Cu (II). The inhibition of biological processes by Cu (II) is more physicochemical and not a biological transport process. This is because sorption plays a crucial role in heavy metal uptake. The sorption rate and internalization of Cu (II) by nitrifiers is faster than that of Zn (II), Ni (II), and Cd (II). Cu (II) also causes a rapid loss of membrane integrity. When Cu (II) enters the cell, it catalyzes the hydroxyl radicals’ production, which results in redox cycling activity which causes stress, thereby causing damage to the membrane functions and affecting the activity of anammox bacteria [29].

### 4.2. Zinc

Zn (II) has also been shown to possess an inhibitory effect on anammox. However, the toxicity of Zn (II) is lower than the toxicity of Cu (II) towards anammox bacteria, with inhibition concentration reported to be 3.9–59.1 mg/L higher than Cu (II) inhibition concentration. Although Zn (II) has a lower inhibition effect than Cu (II), the inhibition caused by 20 mg/L of Zn (II) to anammox sludge and nitrification sludge is irreversible [27]. The ZnO nanoparticle is known to inhibit the biological process by decreasing the number of bacterial cells in the microbial community, thereby decreasing the diversity of microorganisms in the sludge system [32]. However, it is not clear whether the effect of ZnO on the microbial community is caused by Zn^2+^ or the nanomaterials themselves. Thus, Wu et al. [33] investigated the impact of ZnO and Zn^2+^ in nitrification. The result obtained indicated that 0.1 mg/L of Zn enhanced nitrification, while the same concentration of ZnO inhibited nitrification; hence, the authors concluded that the toxicity of ZnO is caused by the nanoparticle itself. Moreover, the author also noted that both Zn nanoparticles and ZnO cause the production of highly reactive oxygen species (ROS) in nitrifiers. The study also showed that the inhibition was linked with the expression of amoA gene rather than the hao and nxrA genes. This confirmed that the difference between Zn ions and ZnO inhibition towards nitrification bacteria is due to ROS production and the expression of amoA.

### 4.3. Cadmium

**Figure 2:**
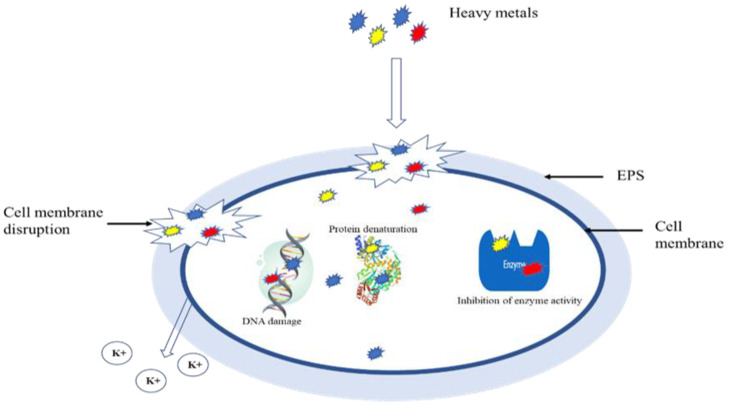
Inhibition of cell growth by toxic heavy metals. Edited from [36,37].

The increase in Cd use in industries results in its accumulation in WWTPs; Cd (II) is widely used in industrial processes i.e., batteries, pigments, chemical stabilizers, and metal coatings. If it is left untreated it can cause damage to human organs. Cd (II) also affects biological processes in WWTPs such as aerobic nitrification, anaerobic denitrification, anammox, and biological phosphate removal [27]. The toxicity of Cd (II) toward biological processes is attributed to its ability to inhibit many significant enzymes for wastewater treatment. Moreover, Cd (II) is also capable of interrupting microbial metabolisms as well as damaging the cell membrane of the bacteria (Figure 2). Nitrifying bacteria are one of the highly sensitive organisms to Cd (II); this is because nitrifiers are sensitive to toxic external factors owing to their low growth rate. Their activity can be inhibited by as low as 1 mM of Cd (II), and this concentration can inhibit up to 70% of ammonium oxidation [8]. 

Li et al. [34] investigated the effect of Cd (II) on enzyme activity and microbial communities in a sequencing batch reactor (SBR). Different concentrations were found to affect the biological system e.g., 10–40 mg/L inhibited COD removal slightly, and 40 mg/L severely inhibited ammonium oxidation. Moreover, the authors also observed that high concentrations of Cd (II) induce the production of reactive oxygen species in activated sludge. This results in the release of extra lactate dehydrogenase, which in turn affects the morphology and physiological functions of microorganisms present in activated sludge. These results are comparable with the findings by Zhang et al. [25]. The authors investigated the impact of cadmium on partial nitrification (PN) in SBR and found that Cd concentration < 5 mg/L did not affect PN. However, increasing concentration to 10 mg/L resulted in 30% decrease in PN efficiency. The authors further investigated the effect of cadmium on AOB and NOB and found that concentrations below 5 mg/L did not affect the activity of AOB and NOB. The pH of wastewater, and the availability of complex organic and inorganic substances in WWTPs is said to link with the toxicity, mobility, and solubility of Cd (II). The cadmium found in the leachate at pH 7.2–8.5 is normally the dissolved cadmium; the solubility of cadmium depends on the pH, and at low pH the solubility increases. Up to 130 mg/L of Cd (II) on municipality leachate has been reported. Although Cd (II) is toxic to the microbial population, there are researchers who have reported microorganisms that are resistant to cadmium. Yamina et al. [35] successfully isolated a bacterial strain that is resistant to Cadmium from hospital wastewater. The isolated strains were characterized and found to be species *Klebsiella pneumoniae* and *Pseudomonas aeruginosa*.

### 4.4. Lead

Pb (II) is toxic to biological wastewater treatment systems. The toxicity of lead is a result of ion displacement or its ability to replace important ions present on cellular sites thereby blocking essential functional groups such as polynucleotide, enzymes, and important nutrient transport systems. Additionally, lead can also denature or inactivate the active conformation of biological molecules such as enzymes. Interruption of membrane integrity has also been reported as a result of high lead concentration [8]. To date, the toxicity of Pb (II) has only been studied in batch reactors, with up to a 50% decrease in the activity of anammox sludges being reported to be inhibited by 10 mg/L of Pb (II) [27]. Although Pb (II) is toxic to many microorganisms present in biological WWTPs, Zhang et al. [8] reported a sulfate-reducing bacteria that was capable of removing up to 99.5% of Pb (II) from wastewater with a 9.2 g/m^3^ loading rate of Pb (II). The author noted that the removed Pb (II) was then deposited into the sludge as PbS. Little Pb (II) was removed, and this ability was attributed to cell adsorption and organic binding.

### 4.5. Nickel

Ni is an essential element for the organisms at micro levels; however, it can be toxic at high concentrations. Ni is especially toxic to biological wastewater treatment systems. However, its effect on anammox is not yet extensively studied [27]. Although Ni (II) is normally present in municipality WWTPs in low concentrations i.e., 0.1 and 0.5 mg/L, there are instances where the concentration gets as high as 10 mg/L as a result of industrial effluent. This excessive concentration affects biomass growth and biological intracellular metabolism (refer to Table 1). It does this by entering the microbial cell and distracting microbial protein function and structure (Figure 2). This can lead to the inhibition of biological systems in the wastewater treatment system. Other studies have observed inhibition of chemical oxygen demand (COD), nitrogen removal by Ni (II), and changes in the diversity and abundance of microorganisms were also noted as a result of Ni (II) [38]. Sun et al. [39] investigated the impact of Ni (II) on EBPR, and the result indicated that 1 mg/L decreased efficiency of phosphorus removal from 99.7% to 38.3%, while a 10 mg/L dose of Ni (II) decreased the efficiency from 99.7% to 0%. Moreover, the authors further investigated the mechanism of inhibition and found that there was no destruction of cell membranes, even though an increase in production of reactive oxygen species was observed. However, the inhibition in the activity of exopolyphosphatase and a decrease in the release of polyphosphate was observed. Furthermore, Ma et al. [38] showed that Ni (II) affected the production of extracellular polymeric substances in the activated sludge. The author further indicated nitrogen removal rate and microbial enzymatic activity were negatively affected by Ni (II) concentration from 10 to 30 mg/L and 5–30 mg/L, respectively.

### 4.6. Arsenic

Arsenic is one of the toxic metalloids that have been shown to harm nitrification and denitrification. The common species of arsenic are arsenite As (III), arsenate As (V), monomethylarsonic acid (MMA), and dimethylarsinic acid (DMA). Among these species As (III) poses the highest toxicity effect on nitrification and denitrification. Although As (III) is toxic to nitrification, it has higher half-maximal inhibitory concentration values (IC50) compared to other heavy metals, with IC50 being 86.6 mg /L compared to Cd (II), Hg (II), Pb (II) and Cr (VI), which have IC50 of 7.0, 2.3, 10.4, and 9.8 mg /L, respectively. Therefore, this indicates that As (III) has lower toxicity compared to other heavy metals [40]. The toxicity of arsenic is related to its oxidation state which affects its bioavailability [41]. However, the inhibition of the anammox process by arsenic has been said to be reversible. Ma et al. [42] reported that high arsenic concentration affects EPS by making it loose thus resulting in elevated permeability which results in an increased probability of direct contact between cells and As (III). Moreover, Papirio et al. [41] indicated As (III) is less toxic at neutral pH; this is due to the oxidation of As (III) to As(V). The authors further indicated that As (V) does not affect nitrification.

## 5. Current Strategies in Place to Prevent Inhibition of Biological Processes by Heavy Metals

To alienate the effect of heavy metals on biological methods in WWTPs, several treatment methods have been explored such as ion exchange, electrochemical methods, and reverse osmosis. However, these methods have been said to have many drawbacks, such as the generation of toxic sludge which requires expensive treatment methods [43]. Additionally, they are not always effective especially when heavy metals are present in higher concentrations. Hence, other environmentally friendly methods have been brought forward [44]. Adsorption has attracted attention owing to its benefits such as being easy to operate, the requirement for less complicated design, and the environmentally friendly method of heavy metal removal. The process of adsorption involves the use of an adsorbent as a solid surface to absorb liquid solute. Several adsorbents such as activated carbons, zeolites, and clay minerals have been successfully used as remedial strategies to pretreat heavy metals from wastewater. The application of environmentally friendly green adsorbents such as biochar has been growing; these green adsorbents are normally made up of straw, bagasse, and shells of peanuts or eggs [45]. However, the separation of this absorbent from treated water is problematic; hence, the use of magnetic nanoparticles has been becoming popular since they address separation problems associated with the use of normal adsorbents [46].

Different ways of synthesizing nanoparticles with magnetic properties have been reported in the literature. The mechanism of heavy metal separation by magnetic nanoparticles involves the application of an external magnetic field [46]. Although magnetic nanoparticles have the potential to replace conventional adsorbents, they have drawbacks. For example, they oxidize in the air, which can lead to loss of their magnetism; surface leaching of magnetic nanoparticles occurs, which leads to difficulties in separation. There has been ongoing research that seeks to address these problems, and surface modification has emerged as a promising strategy to overcome these limitations. However, surface modification has been said to increase the operational cost of nanoparticles; hence, some researchers have proposed mixing magnetic nanoparticles with another nanoparticle to avoid modification of nanoparticles. The use of multiple nanoparticles only addresses the issue of modification but not the cost, since purchasing or production of multiple nanoparticles may be costly. Therefore, the cost of nanoparticles remains the stumbling block in their application. Nanotechnology generally is expensive, therefore its application in wastewater treatment is not financially sustainable, especially for economically struggling countries. Mpongwana and Rathilal [47] have shown that nanotechnology is expensive technology as compared to conventional methods used in WWTPs for heavy metal removal. Several approaches have been recommended to reduce the cost of nanotechnology in WWTPs.

Nanoparticle reuse and regeneration have emerged as promising approaches to reducing the costs of nanoparticle application in WWTPs. However, this approach requires good separation, where separation of nanoparticles is problematic due to leaching. Additionally, the synthesis of nanoparticles with cost-effective raw materials such as plant materials and biological synthesis of nanoparticles has been shown to reduce the cost of nanoparticle production, subsequently reducing the cost of their application in WWTPs. Moreover, the use of bacteria, algae, fungi, and yeasts as bio-absorbents has been explored as a cost-effective method of removing heavy metals from WWTPs. The removal of heavy metals by the biosorption process involves the binding of heavy metals to live microbial cells or inert biomass. This process is efficient in the removal of heavy metals; however, the design and operation of the system are crucial for the success of this process [44]. However, the application of microorganisms as an adsorbent was ridiculed owing to the toxic compounds found in the effluent because of hurdles in biomass separation.

The poor separation can also result in the production of a large amount of sludge and the inability to be recycled for re-utilization. Some researchers have investigated ways of addressing this issue. The application of magnetotactic bacteria (MTB) has emerged as a promising solution to this problem. MTB was shown to be effective candidate adsorbents for heavy metal removal. MTB can migrate towards the applied magnetic field. This characteristic makes it easy to separate them from the effluent and reuse them [48]. Other drawbacks of biosorption have been reported like the low adsorption efficiency for wastewater containing high concentrations of heavy metals. However, several strategies have been reported to improve biosorption. Chemical surface modification and internal resistance modification are some of the approaches that have been shown to improve biosorption. The modification of biomass surface is carried out by four methods which are physical adsorption, microprecipitation, ion exchange, and surface complexation. Another recommended approach to improve biosorption is the immobilization of biomass, which can also improve better renewability, combining the biosorbent with another adsorbent [49].

Other absorbents such as hydrogels have also shown to be effective for heavy metal removal. Other technologies such as membrane filtration, photocatalysis, etc., have also been investigated for heavy metal removal [50]. Due to the challenges mentioned in this section regarding pretreatment methods of heavy metals, the pretreatment is only applied in case of toxicant introduction to the WWTP; however, the unexpected introduction of heavy metals in WWTPs may result in the introduction of heavy metals into the biological systems, resulting in inhibition of biological processes, which may necessitate effective recovery strategies to recover biological processes in speed time.

## 6. Recovery Strategy for the Biological Wastewater Treatment Process

### 6.1. Physical Methods to Recover Biological Wastewater Treatment

There are pretreatment steps that can be incorporated in the case of heavy metal loadings in WWTPs; unfortunately, there are instances where there are unexpected loadings which lead to the entrance of heavy metals into biological wastewater treatment systems. The conventional methods discussed in the section above (refer to Section 5) are sometimes applied as a recovery strategy in the case where there’s an unexpected introduction of heavy metals into the biological system. Application of the physical and chemical methods that are normally used for pretreatment is tricky since these methods produce toxic byproducts which may result in further inhibition of the microbial population responsible for biological processes. Moreover, there are other disadvantages to these methods which makes them less attractive, particularly for the recovery of biological processes, such as high investment and facility idling [9]. Moreover, conventional methods are not effective for all heavy metals. For example, it has been said that conventional methods are not effective in the recovery of biological processes inhibited by cadmium [8]. Thus, it has been suggested that sustainable ways and efficient methods that will recover the process without causing additional harm to the microbial community need to be investigated. The important aspect of process recovery is the time it takes to recover the process and the viability of the process after the recovery. Several methods have been proposed for the recovery of nitrogen removal after inhibition by heavy metals. This section will critically review these methods.

### 6.2. Recovering of Anammox Using a Chelating Agent

Understanding the inhibition mechanism is important for developing a successful remedial strategy for the recovery of biological processes [31]. Several recovery strategies for the biological process have been investigated. Washing of sludge with buffer and EDTA has been shown to be one of the most efficient ways of recovering inhibited biological processes. In this process, heavy metals that are absorbed in the cell surface are removed by oscillation and centrifugation. This process involves the addition of EDTA. This promotes the binding of extracellular polymeric substances (EPS) to the heavy metal, and subsequently transforms into EDTA-meta complex that stimulates outward heavy metal diffusion into the granular sludge. During the recovery, it is important to decrease the substrate loading because the heavy metal causes a decrease of anammox–substrate inhibition. Li et al. [51] successfully recovered 98.49%, 95.61%, 97.42%, and 93.25% sludge activity which was inhibited by 2.5, 5, 10, and 25 mg/L Ni (II) respectively. Although EDTA is effective for the recovery of anammox inhibited by many heavy metals, this strategy is only effective for cationic metals that are capable of bonding with OH to form precipitation. This strategy is not always effective for heavy metals that exist as anionic metals. As an example, EDTA cannot recover Cr (II)-inhibited anammox because Cr (II) only exists as CrO_4_^2−^. Additionally, EDTA has been reported to cause additional toxicity to nitrification bacteria, thereby causing damage to the nitrification process [8]. 

Hydroxylamine (NH_2_OH) can also be used to recover inhibited anammox because it can stimulate anammox bacteria. NH_2_OH is known to be an important intermediate of anammox because it increases the rate of total nitrogen, plays a significant role in the restoration of NO_2_ inhibition, and can reduce Cr (VI) to Cr (III). NH_2_OH is normally used for the recovery of anammox inhibited by heavy metals that exist as anionic metals. It has been shown that Cr (VI) can be relieved by the addition of 1.83~9.17 mg N/L NH_2_OH. This happens due to the reduction of extracellular Cr (III). The mechanism by which NH_2_OH relieves inhibition of anammox by Cr (VI) occurs in two pathways, which are the reduction and strengthening of anammox through stimulation [52]. Other chelating agents such as nitriloacetic acid (NTA) and diethylenetriamine penta acetic acid (DTPA) can be used to recover anammox since they can form metal–ligand complexes. Although these chelating agents are effective for recovering anammox, they are not however widely used due to their disadvantages for example NTA has been identified as a class II carcinogen [29]. EDTA sometimes is combined with other strategies to work efficiently. For example, it can be mixed with bio-stimulation through low-intensity ultrasound, or form a complex with S_2_ deactivation, or first wash the sludge with EDTA and Ca^2+^ regulation. Li et al. [53] reported an innovative strategy to recover a Fe-inhibited anammox. Where EDTA was combined with betaine, this strategy was proven to accelerate anammox recovery.

#### Post-Treatment of Chelating-Metal Complex By-Products

Chelating agents work by forming a complex with metals within the bacterial cell, thereby promoting the outward movement of heavy metals. The chelating agent–metal complex that results from the application of a chelating agent becomes toxic and therefore may require additional treatment. EDTA is the most used chelating agent for recovery inhibiting total nitrogen removal. The metal–EDTA complex is toxic and difficult to treat from wastewater through conventional precipitation processes. Additionally, the metal–EDTA complex cannot be used by microorganisms in the biological system. This is due to the inhibition effect of EDTA on the adsorption process of heavy metals by biosorbents and sorbents [54]. Several methods have been investigated for their ability to treat metal–EDTA-contaminated wastewater such as Fenton/Fenton-like reaction-hydroxide precipitation (FR-HP), interior microelectrolysis (IM), photocatalytic oxidation, and membrane filtration [55]. However, most of these methods are not practical in large-scale wastewater treatment. Fenton has been gaining popularity in the treatment of metal–EDTA-contaminated wastewater. Fenton is an advanced oxidation process that has been used to treat electroplating industrial effluent; this process works by de-complexing EDTA from metal, followed by decomposition of an organic compound. Fenton generates hydroxyl radicals from the mixing of ferrous ions and H_2_O_2_; the generated hydroxyl radicals have strong oxidizing properties which are capable of oxidizing organic–metals compounds into inorganic forms [55]. Recently studies had proposed a combination of Fenton with other conventional methods to increase its effectiveness. Nguyen et al. [56] proved that combining Fenton with ozonation improves the effectiveness of treatment. Although several methods such as Fenton have been proven to be effective for the treatment of chelating agent-metal complex, the toxic by-product produced during these methods makes them impractical for direct application to biological wastewater treatment systems since they may cause further damage to recovering microbial population. Therefore, non-toxic methods of treating chelating agent–metal complex that results from the application of chelating agents for recovery of biological treatments still need to be investigated.

### 6.3. Recovering of Anammox Using External Field Energy

External field energy has been used to enhance anammox; several studies have reported different external field energy which can enhance anammox biomass activity such as magnetic fields, external electrostatic, and low-intensity ultrasound. Electrostatic fields have been employed in biological research for decades for different reasons such as enhancing cell growth, cell death, repair of genes, gene transfer, diagnostics, and sensing devices. Application of electrostatic field to microbial cells causes polarization of the cell membrane which results in osmotic imbalance and permeability of the cell membrane. This provides a possibility to create mechanical instability in the membrane of microbial cells because of increased membrane permeability induced by critical membrane potential. In addition to that, the pulsed electrostatic field can also enhance enzyme activity, particularly those that have heme. It has been therefore hypothesized that anammox is enhanced because of a mass transfer of anammox cells, which causes changes in the morphology of the membrane when the low-level electrostatic field is applied. Thus, promoting key enzymes through conformational change and faster transfer of heme [57]. 

The growth of microorganisms under anaerobic conditions has been reported to result from various redox potentials between electron donors and acceptors, thereby enhancing the metabolism of the microbial cell [58]. This can result in enhanced total nitrogen removal, even when the concentration of biomass is low [59]. Ultrasound has also been reported to enhance anammox in various studies [29]; ultrasound is preferred because of its low operation cost. This is due to its ability to last for several days after its application [29]. Other studies have reported that the effect of ultrasound lasts up to 6 months after being applied to anammox. Other benefits of applying external field energy in the biological system include the expression of proteins, DNA synthesis, 16 RNA transcription, synthesis of ATP, and promotion of metabolic activity [60]. Zhang et al. [61] show that electric power of 1.5 V enhanced anammox bacterial activity by enhancing the rate of ion migration and the permeability of membrane in an inhibited anammox biomass. Moreover, Qiao et al. [58] reported the recovery of anammox biomass in 2 weeks after inhibition by heavy metals using a 2 V/cm electric field. These results are comparable to the findings of Cheng et al. [62] who reported a reduction of cell lysis caused by tetracycline stress using a 3 V electric field. Ultrasound irradiation has also been shown to recover inhibited anammox activity. Zhang et al. [29] reported that at 28-kHz ultrasound intensity and exposure time of 0.7 W cm^−2^ and 1.9 min, respectively increased the nitrogen removal rate by 0.1971 kgN m^3^d^2^.

### 6.4. Recovering of Anammox Using Biological Accelerants

Biological accelerants have been investigated for their ability to recover inhibited anammox. They are the preferred strategy to recover or enhance biological nitrogen removal since they are deemed to be environmentally friendly. Biological accelerants that are normally used for recovery of anammox contain growth factors; these growth factors are required by the microorganism in small amounts, and they are needed by microorganisms for the assimilation and catabolism process [9]. It is important to note that microorganisms are not able to synthesize these growth factors to meet their growth needs. Biotin (vitamin H), Flavin adenine dinucleotide (FAD), cytokinin, and L-cysteine have been shown to have excellent capability to promote biological nitrogen removal [63]. All these biological accelerants play a crucial role in cell metabolism. Biotin is known as a coenzyme of several carboxylases; thus, it is important for cell metabolism of fats and proteins. Biotin can enhance the removal of chemical oxygen demand (COD) and biochemical oxygen demand (BOD). FAD is important for the rapid growth of the microbial cell; this is because FAD is crucial for numerous redox reactions in vivo which are important for the enhancement of cell growth. 

Cytokinin is vital for several biological processes in the microbial cell such as accelerating microbial activity restoration, enhancing microbial cell division, and inducing the formation of buds, thereby promoting microbial growth. L-cysteine is responsible for cell reduction and metabolism of phospholipid that occurs in the liver and is responsible for reducing liver cell damage and enhancing the recovery of liver functioning [7]. Moreover, L-cysteine can also act as an antidote for the poisoning of bacterial cells with heavy metals. 

Quinoid redox mediators are another biological accelerator that has been shown to play an important role in promoting biological nitrogen removal. Redox mediators act as transport to promote the electron transfer process between the donors and acceptors. Quinone redox mediators were shown to accelerate the transport of electrons. Additionally, sodium humate (HA) and riboflavin (VB2) have also been shown to improve the electron transport activity of denitrification, particularly the electron transport activity for nitrite removal [63]. 

Table 2 shows a comparison between different recovery mechanisms. Wang et al. [9] show 100% recovery of Cu-inhibited nitrogen removal within 8 days. This is a shorter recovery time compared to the recovery time which was reported by Zhang et al. [29], who reported a 100% recovery of Cu-inhibited nitrogen removal in 64 days when a combination of chelation, bio-accelerator, and low-intensity ultrasound was applied. This could be attributed to the negative effect chelation may have on the acquisition of important metals required for bacterial growth. Wang et al. [64] compared the application of bio-accelerators only, and a combination of bio-accelerators and redox mediators for Cr (II) inhibited the denitrification process. The authors find out that the application of a combination of L-cysteine, flavin adenine dinucleotide (FAD), biotin, cytokinin, and redox mediators is superior in the recovery of denitrification, with over 90% being recovered in less than 40 T. Whereas, the application of L-cysteine, flavin adenine dinucleotide (FAD), biotin and cytokinin only recovered 90% of the denitrification process in 42 T. This showed that redox mediators boasted the recovery of denitrification further. These studies showed that, though not in all cases, a combination of strategies will work well in the recovery of biological processes. Understanding the inhibition mechanism of a particular heavy metal may be the key to developing a recovery strategy that is effective to recover biological processes in a shorter time.

### 6.5. Post-Treatment of Biological Accelerants By-Products

Although no toxic by-products have been reported when bio-accelarators are employed to recover biological processes, it is important to note other unintended negative consequence that needs to be looked at. Protozoa is one of the microorganisms present in the wastewater treatment system. The role of protozoa in wastewater has been studied, and it was shown that their primary role was to clarify the effluent [6]; additionally, protozoa is known to prey on suspended bacteria. As a result, protozoa have been shown to affect the diversity of the microbial population in the wastewater treatment plant, although it was said that biofilm is resistant to protozoa predation. It has been reported that protozoa affect biofilms significantly, and that the presence of protozoa reduces the abundance of AOB and anammox bacteria, thus reducing nitrification to up to 50% [65]. The growth of several protozoa species has been said to be enhanced by vitamins [66]. Therefore, the addition of these growth factors for recovery of the biological process may enhance the growth of protozoa thus increasing predation, leading to impairment of nitrification. Therefore, the effect of these growth factors on protozoa and the subsequent effect on nitrification and denitrification needs to be investigated [67]. This will enable the development of suitable strategies to monitor protozoa, thus minimizing unexpected drastic growth of protozoa in the biological system.

## 7. Drawbacks of These Recovery Strategies

The recovering of biological process is essential since restarting the process may result in a long startup time, thus increasing operational costs. Although these recovery strategies are effective for recovering inhibited biological wastewater treatment, they also have drawbacks that prohibit them from being applied in large-scale wastewater treatment systems. The physical method only removes the heavy metals in the system; however, it does not catalyze the regrowth of the microorganism. Moreover, the physical method may produce toxic byproducts that can be detrimental to microbial metabolism [68]. This may result in further inhibition of the biological process. Additionally, the physical methods are not effective for low concentrations of heavy metals [44]. For example, chemical precipitation produces metal hydroxides, carbonates, and sulfides [69]. 

High sulfide concentrations have been shown to prohibit the growth of certain microorganisms, causing depolarization of the membrane, damage to DNA, lipid peroxidation, denaturation of protein, and lower ATP levels. Additionally, sulfide may inhibit the superoxide dismutase and catalase, which is important for cellular defense against oxidative stress. Inhibition of these enzymes may result in a raise in glutathione levels and ROS [70]. 

The use of a chelating agent has also been investigated. EDTA is one of the most popular chelating agents; although EDTA has been highly investigated for its ability to recover biological wastewater treatment processes, some studies have reported that it may be detrimental to the nitrification process [8]. Chelating agents interfere with the ability of a microorganism to acquire metals and bioavailability for essential processes. Therefore, the essential metal metabolism of microorganisms may be affected. It has been reported that chelation inhibits biological processes that require metal-dependent proteins such as metalloproteases and transcription factors. This may negatively affect the homeostasis of the microorganism, thereby affecting microbial nutrition acquisition, growth of microorganisms, and adhesion to biotic and abiotic structures [71]. Furthermore, low concentrations of EDTA have been shown to inhibit the adhesion of biofilm, thereby preventing biofilm formation, and reducing proliferation and biofilm colonization [72]. 

Hydroxylamine (NH_2_OH) is one of the anammox metabolites used for recovery of heavy metal inhibited biological wastewater treatment processes. NH_2_OH has been reported as a toxic metabolite to anammox bacteria. NH_2_OH has been said to inhibit hydrazine dehydrogenase (HDH), an enzyme known to be responsible for N_2_H_4_ oxidation to N_2_. This results in a reduction of anammox activity. The hydroxylamine tolerance for NH_2_OH in anammox bacteria has been shown to range between 60 and 70 mg N/L for 4 g/L [73]. Additionally, Huang et al. [74] reported that dosing with NH_2_OH suppressed NOBs. It was postulated that NOB was suppressed by NO, which was observed accumulating when NH_2_OH was added, and the production of NO was due to oxidation of NH_2_OH. These results are comparable to the result obtained by Sui et al. [75] who also reported that NH_2_OH dosing inhibited NOB activity.

## 8. Recent Work on Recovery Strategies

In previous years, the recovery included removal of heavy metals from the sludge using different methods such as either physical methods or washing with EDTA; following the self-recovery, however, this strategy was found to be time-consuming. Hence, other methods that stimulate microbial growth have been employed, such as the use of biological accelerants. Recently the combination of physical methods, application of chelating agent, and biological accelerants at the same time has attracted interest since it was shown to recover anammox faster [29]. Wang et al. [64] investigated the application of a bio-accelerator, combined with a redox mediator, for recovery of the denitrification process. The bio-accelerator used for this study included a combination of L-cysteine, flavin adenine dinucleotide (FAD), biotin, and cytokinin. The result obtained from this study indicated that bio-promoters only accelerated recovery; however, the introduction of bio-promoter accelerated the recovery even further. Therefore, the authors deduced that a combination of bio-promoters and redox mediators can recover the abundance of nirS-type and napA-type denitrifiers. 

Recently, there have been studies reporting the recovery of anammox from bioavailable metal inhibition such as Fe (II); this is because the inhibition mechanism of non-bioavailable metals differs from that of bioavailable heavy metals, therefore it has been reported that bioavailable metals require different recovery strategies than those used for non-bioavailable heavy metals. Li et al. [53] reported a novel recovery strategy for bioavailable metal Fe (II). The strategy involves the application of chelating agents (EDTA-2Na and betaine). These chelating agents were chosen for their ability to accelerate the disinhibition of Fe (II); the results found from this study indicated that 0.5, 1.0, 1.5 and 2.0 mM of EDTA-2Na did not affect the recovery of anammox; however, 2.0 mM of betaine accelerated the recovery of anammox. Nevertheless, the addition of betaine for a prolonged period resulted in a reduction of anammox activity. NH_2_OH has also been gaining popularity in the recovery of anammox; NH_2_OH is one of the significant intermediates of anammox, and it plays a crucial role in the utilization of substrate and anammox transformation. NH_2_OH is especially important in increasing the rate of nitrogen removal, restoration of NO^2−^ inhibition, and reduction of Cr (VI) to Cr (III); it has been shown that only 1.27 mgN/L NH_2_OH can recover anammox in one day [52]. Huang et al. [74] reported restoration of partial nitritation anammox by NH_2_OH; the result obtained from this study indicated that partial nitrification was recovered in 5 days by dosing with 10 mg N/L of hydroxylamine. Moreover, Sui et al. [75] reported that NH_2_OH performed better in the recovery of partial nitritation anammox compared to hydrazine (N_2_H_4_). The authors further indicated that acceleration of ammonia oxidation by NH_2_OH was due to enhancement of hao genes, which in turn enhances the recovery of partial nitritation anammox. 

Phosphate buffer has also been used to recover inhibition of denitrification by Cu^2+^. Phosphate buffers prevent an increase in pH, thereby reducing bio-accessibility and solubility of Cu^2+^ by 40–69%. Ma et al. [76] investigated the potential of recovering denitrification using phosphate, and the authors found that phosphate recovered 99% of nitrogen removal efficiency that was inhibited by Cu^2+^ in 10 days. Additionally, phosphate also restored enzyme activity to its original levels. Biological accelerant has also been gaining popularity due to their benefits such as environmental friendliness, and their exertion of little toxicity on the microbial population. Some of the popular bio-accelerants that have been highly explored include Biotin, (FAD), cytokinin, and L-cysteine [7,8].

## 9. Conclusions

Recovery of inhibited biological processes is important in wastewater treatment plants; this is because, at times, the unexpected introduction of heavy metals may result in inhibition of biological processes that are crucial for wastewater treatment. The addition of fresh sludge may be costly; therefore, it may not even be an option for developing countries. Different methods of recovering biological processes inhibited by heavy metals have been reported. The application of EDTA, bio-accelerators and external fields have been popular methods owing to reports discrediting the application of physiochemical methods due to toxic byproducts that may cause further harm to the important microbial community. However, the application of a chelating agent may impair the ability of a microorganism to uptake heavy metals that are important for other metabolic processes. Furthermore, the application of EDTA has been shown to affect the nitrification process. Although bio-accelerators have emerged as a promising strategy for the recovery of heavy metals, a lot of work still needs to be done such as engineering microorganisms to produce bio-accelerator to meet their growth needs, developing a chelating agent that can target specific metals of interest, and investigating the effects these recovery strategies have on the diversity of the microbial population present in wastewater.

## Figures and Tables

**Table 1:** Heavy metals and their inhibition effect on wastewater treatment biological processes.

Reference	Heavy Metal	Inhibited Process	Metal-Inhibition Conc	Percent Inhibited	IC_50_
**Ochoa-Herrera et al. [28]**	Cu (II)	denitrification	50 mg/L	100%	0.95 mg/L
**Zhang et al. [8]**	Cu (II)	anammox	16.3 mg/L	20.1%	30 mg/L
**Zhang et al. [8]**	Zn (II)	anammox	20.0 mg/L	20.1%	25 mg/L
**Kimura and Isaka, [23]**	Ni	anammox	10 mg/L	87%	-
**Kimura and Isaka [23]**	Co	anammox	12 mg/L	64%	-
**Kimura and Isaka [23]**	Zn	anammox	15 mg/L	79%	-
**Buaisha et al. [30]**	Cu (II)	heterotrophic bacteria concentration	0.7 mg/L	25.00%	-
**Buaisha et al. [30]**	cadmium	heterotrophic bacteria concentration	0.7 mg/L	8.76%	-
**Bi et al., [31]**	Cd	anammox biomass	-	-	11.16 ± 0.42 mg/L
**Bi et al. [31]**	Ag	anammox biomass	-	-	11.52 ± 0.49 mg/L
**Bi et al. [31]**	Hg	anammox biomass	-	-	60.35 ± 2.47 mg/L
**Wang et al. [9]**	Cu^2+^	nitrification bioactivity	50.00 mg/L	80.00%	-

**Table 2:** Comparison of different recovery strategies for recovery of heavy metal inhibited biological processes.

Ref	Inhibited Process	Inhibiting Metal	Method of Acceleration	Accelerant	Percent Recovered	Recovery Time	Recovery Cycle
**Wang et al. [9]**	AOB and NOB	copper	Bio-accelerators	biotin, l-aspartic acid and cytokinin, l-aspartic acid	100% recovered	8 days	-
**Zhang et** **al. [29]**	nitrogen removal rate	copper	chelating + bio-accelerator+ External field energy	EDTA + biostimulation+ low-intensity ultrasound	100% recovered	64 days	-
**Zhang et al. [29]**	dehydrogenase activity	copper	chelating + bio-accelerator+ External field energy	EDTA + biostimulation+ low-intensity ultrasound	100% recovered	110 days	-
**Wang et al. [8]**	nitrifying bacteria	cadmium	bio-accelerators	Biotin, l-aspartic acid, and cytokinin	100% recovered	7 days	-
**Wang et al** **. [64]**	denitrification	Cr (VI)	natural conditions	natural conditions	89.28 ± 0.20%	-	52 T
**Wang et al. [64]**	denitrification	Cr (VI)	redox mediators + bio-promoters	L-cysteine, flavin adenine dinucleotide (FAD), biotin, cytokinin, and redox mediators	<90.00%	-	>40 T
**Wang et al. [64]**	denitrification	Cr (VI)	redox mediators + bio-promoters	L-cysteine, FAD, biotin, and cytokinin	90.3%	-	42 T
**Wang et al. [64]**	denitrification	Cr (VI)	natural conditions	natural conditions		-	63 T

## Data Availability

Not applicable.
